# Nuclear mRNA Degradation Pathway(s) Are Implicated in *Xist* Regulation and X Chromosome Inactivation 

**DOI:** 10.1371/journal.pgen.0020094

**Published:** 2006-06-16

**Authors:** Constance Ciaudo, Agnès Bourdet, Michel Cohen-Tannoudji, Harry C Dietz, Claire Rougeulle, Philip Avner

**Affiliations:** 1 Unité de Génétique Moléculaire Murine, Institut Pasteur, Paris, France; 2 Unité de Biologie du Développement, Institut Pasteur, Paris, France; 3 Institute of Genetic Medicine and Howard Hughes Medical Institute, Johns Hopkins University School of Medicine, Baltimore, Maryland, United States of America; The Babraham Institute, United Kingdom

## Abstract

A critical step in X-chromosome inactivation (XCI), which results in the dosage compensation of X-linked gene expression in mammals, is the coating of the presumptive inactive X chromosome by the large noncoding *Xist* RNA, which then leads to the recruitment of other factors essential for the heterochromatinisation of the inactive X and its transcriptional silencing. In an approach aimed at identifying genes implicated in the X-inactivation process by comparative transcriptional profiling of female and male mouse gastrula, we identified the *Eif1* gene involved in translation initiation and RNA degradation. We show here that female embryonic stem cell lines, silenced by RNA interference for the *Eif1* gene, are unable to form *Xist* RNA domains upon differentiation and fail to undergo X-inactivation. To probe further an effect involving RNA degradation pathways, the inhibition by RNA interference of *Rent1,* a factor essential for nonsense-mediated decay and *Exosc10,* a specific nuclear component of the exosome, was analysed and shown to similarly impair *Xist* upregulation and XCI. In *Eif1-, Rent1-,* and *Exosc10*-interfered clones, *Xist* spliced form(s) are strongly downregulated, while the levels of unspliced form(s) of *Xist* and the stability of *Xist* RNA remain comparable to that of the control cell lines. Our data suggests a role for mRNA nuclear degradation pathways in the critical regulation of spliced *Xist* mRNA levels and the onset of the X-inactivation process.

## Synopsis

In mammals, each cell of the female contains two X chromosomes and hence, potentially a double dose of all X-linked genes when compared to XY males, who carry a single X chromosome. X-inactivation is the mechanism that ensures the dosage-compensation of X-linked gene products between the two sexes. X-inactivation is under the control of a specific region of the X chromosome, the X inactivation center *(Xic),* which contains the *Xist* gene encoding a large noncoding RNA transcript whose upregulation is critical to the initiation of X-inactivation. Such changes in steady-state transcript level could be due to altered rates of transcription or changes in the stability and processing of the transcript. How expression of *Xist* RNA is regulated and the nature of the mechanisms, which lead to *Xist* upregulation, remain unanswered or only partially answered questions of major importance to the field. In the following article, the authors identify three genes, *Eif1, Rent1,* and *Exosc10,* involved in nuclear mRNA degradation pathway(s), which are required for *Xist* expression upregulation and associated X-inactivation. Inhibition of the function of one or other of these genes leads to a failure of the female cells to undergo X inactivation, suggesting that post-transcriptional nuclear mRNA degradation pathway(s) are essential for the regulation of *Xist* RNA metabolism and X chromosome inactivation process.

## Introduction

Since upregulation of the noncoding *Xist* RNA is concomitant with the establishment of the onset of X-inactivation [1,2], the precise regulation of *Xist* transcript levels in the nucleus is likely to be critical to this process. The increase in steady-state levels of *Xist* with the onset of X-inactivation was thought to be solely dependent on stabilisation of *Xist* transcripts and therefore essentially post-transcriptional. More recently, evidence has accumulated that control at the transcriptional level, mediated by chromatin remodelling at the *Xist* locus induced by biallelic *Tsix* antisense transcription, is of critical importance [3–5] (Navarro et al., unpublished data).

In female embryonic stem (ES) cells—an extensively used ex vivo model for the study of random X-inactivation [6], the biallelic expression of *Xist* and its antisense *Tsix* present in undifferentiated cells can be visualised as pinpoints by RNA–fluorescence in situ hybridization (RNA-FISH) [7]. When initiation of X-inactivation is induced in these cells by differentiation, upregulation of the *Xist* transcript levels on the presumptive inactive X [1,2] is visualised as the formation of a *Xist* RNA domain coating the inactive X, and this occurs concomitantly with the extinction of the antisense *Tsix* signal [8,9]. These initial events are followed by the dynamic recruitment of multiple chromatin modifications to the inactive X, involving polycomb group proteins, extensive histone modifications, and DNA methylation, resulting in the formation of the highly stable inactive heterochromatinised structure which characterises the inactive X [10].

As an approach to identifying additional molecular species that might be implicated in X chromosome inactivation (XCI), we have applied the serial analysis of gene expression (SAGE) technique [11] to the study of male and female 6.5 d post-coitum mouse embryos in its downsized version, which makes possible the exploitation of small samples (SAGE adaptation on downsized extracts [SADE], [12]). At this developmental stage, random X-inactivation has just occurred in the epiblast of female embryos and sexual dimorphism between XX and XY embryos is limited. We reasoned that if there were differences in transcript frequencies between female and male embryos at this stage, a subset of such differences might be implicated in X-inactivation. More than 214 genes overexpressed in female compared with male were identified [13], and a subset was validated using the ES cell system. Of the candidates falling within the category of upregulated genes and which are implicated in RNA metabolism and processing, the *Eif1* (other name, *Sui1-rs1*) gene was retained for functional analysis. *Eif1* (eukaryotic initiation factor 1) plays a critical role in stringent AUG selection during eukaryotic translation [14]. Studies in yeast have demonstrated that mutations in the *Eif1* gene induced a stabilization of transcripts which contain a premature stop codon, indicating that the mutation of this gene can affect both translation and nonsense-mediated mRNA decay (NMD) pathways [15]. The NMD pathway's major role is currently thought to be to proofread mRNAs and to ensure the degradation of mRNAs that contain premature stop codons [16,17]. Protein products of the *Rent1* (other name, *Upf1*), *Upf2, Upf3A,* and *Upf3B* genes are also essential for NMD activity [18].

Here we report that the downregulation by RNA interference of several genes (*Eif1, Rent1,* and *Exosc10* genes) involved in mRNA degradation pathways leads to downregulation of spliced *Xist* transcript production and blocks the onset of the X-inactivation process. Our results suggest that the NMD pathway may play a role in the post-transcriptional regulation of *Xist* metabolism.

## Results

### Generation of Stable Cell Lines Interfered for *Eif1* and *Rent1*

**Figure 1:**
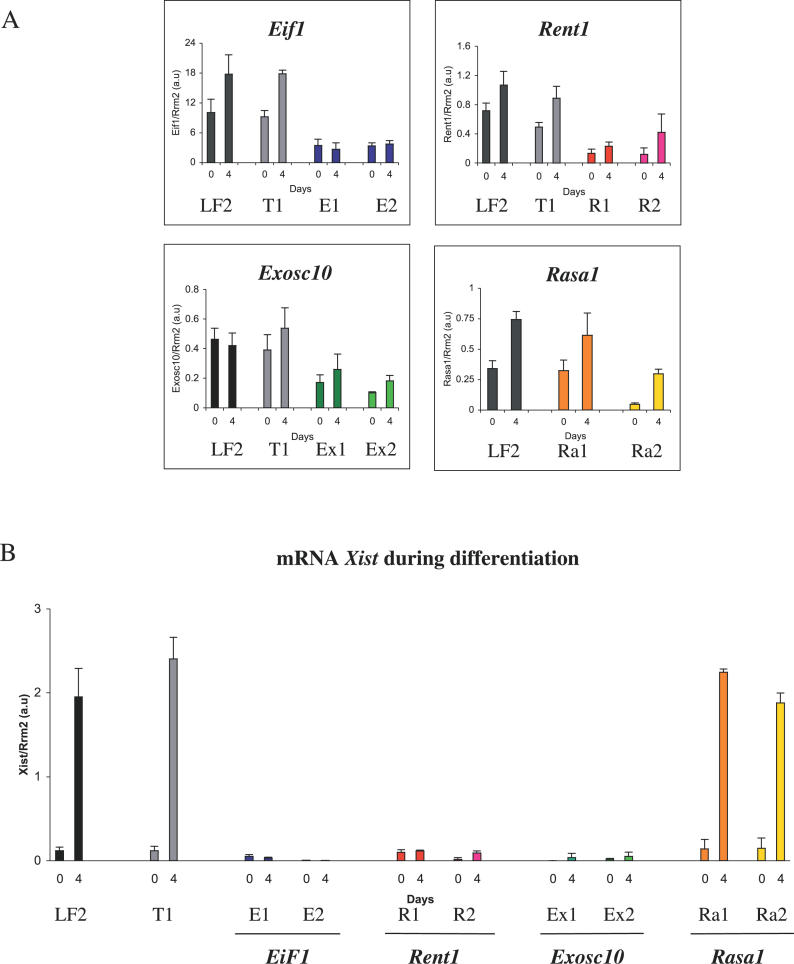
The *Eif1*, *Rent1,* and *Exosc10* Genes Are Essential for the Upregulation of *Xist* mRNA During Differentiation (A) Quantification of *Eif1, Rent1, Exosc10,* and *Rasa1* RNAs using Eif1Up/Lo, Rent1Up/Lo, Exosc10Up/Lo, and Rasa1Up/Lo primers, respectively, at d 0 and d 4 of retinoic acid–induced ES cell differentiation. LF2 is the parental female ES cell line. The T1 clone is a control generated by integration of an empty pSuper-puro vector into the LF2 cell line. The E1 and E2 clones were generated by integration of a pSuper-puro vector containing a hairpin designed against the ORF of the *Eif1* gene. The R1 and R2 clones were generated by integrating a pSuper-puro vector containing hairpins 1 and 2, respectively, directed against the ORF of *Rent1* gene. The Ex1 and Ex2 clones were generated by integrating a pSuper-puro vector containing hairpins 1 and 2, respectively, directed against the ORF of the *Exosc10* gene. The Ra1 and Ra2 clones were generated using the plasmid previously described by Rossant and colleagues [33] that contains a hairpin directed against the *Rasa1* gene. (B) Quantification, using exon1–exon3 primers, of spliced *Xist* RNAs at d 0 and d 4 of differentiation, in the parental female ES cell line (LF2), the T1 control clone, and clones interfered for *Eif1* (E1 and E2), *Rent1* (R1 and R2), *Exosc10* (Ex1 and Ex2), and *Rasa1* (Ra1 and Ra2). Columns and bars show the mean ± standard deviation (SD) (n = 3), expressed in arbitrary units, of the quantity of *Eif1, Rent1, Exosc10, Rasa1,* and *Xist* transcript standardized over *Rrm2* RNA.

In order to investigate the putative role of *Eif1* in particular and the NMD pathway in general in the XCI process, we have exploited RNA interference to generate ES cell lines stably interfered for the *Eif1* and *Rent1* genes [19]. ES clones derived from the female cell line LF2 in which the downregulation of *Eif1* and *Rent1* genes was greater than 70% (E1 and E2 for *Eif1* and R1 and R2 for *Rent1*) were used to characterise the stability of *Eif1* and the *Rent1* interference during ES cell differentiation (Figure 1A). The T1 clone is a control generated by integration of an empty pSuper-puro vector into the LF2 cell line. Quantitative RT-PCR (Q-RT-PCR) analysis of two independent knockdown clones for both *Eif1* and *Rent1* showed that the *Eif1* and *Rent1* expression levels are stably interfered with, both prior to and during ES cell differentiation, at d 4 (Figure 1A) and d 14 of differentiation (unpublished data).

### Impact of Interference on the *Xist* mRNA Level

We monitored the expression of *Xist* using Q-RT-PCR in ES clones stably silenced for *Eif1* and *Rent1* expression, both before and after the induction of differentiation. As shown in Figure 1B, undifferentiated ES cells silenced for the *Eif1* and *Rent1* genes displayed reduced levels of *Xist* RNA compared to control ES cells (LF2, T1). Most strikingly and in contrast to wild-type (WT) LF2 cells, *Xist* expression failed to undergo upregulation during the differentiation of the *Eif1* and *Rent1* silenced clones (Figure 1B). To investigate the specificity of the *Eif1* and *Rent1* interference on *Xist* RNA, we generated stably silenced clones against *Rasa1,* a gene not known to be involved in mRNA degradation or XCI (Figure 1A). Since *Xist* expression was not affected in clones silenced for the *Rasa1* gene (Figure 1B), we conclude that the effects of *Eif1* and *Rent1* knockdown on *Xist* RNA metabolism and inactivation are specific.

### Upregulation of *Eif1* Induced High Levels of *Xist* RNA

**Figure 2:**
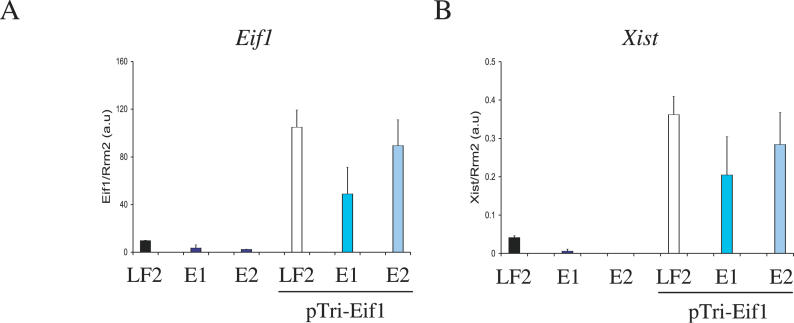
Effect of *Eif1* Upregulation on *Xist* mRNA Levels Quantification of *Eif1* (A) and *Xist* (B) RNAs in undifferentiated parental female ES cell line (LF2), clones knocked down for *Eif1,* and clones lipofected with the pTriEx-Eif1 expression plasmid (see Methods and Materials) after 4 d of selection on G418-containing medium. Columns and bars show the mean ± SD (n = 3), expressed in arbitrary units, of the quantity of *Eif1* and *Xist* transcripts standardized over *Rrm2* RNA.

Further confirmation of the specificity of the *Eif1* interference effect on *Xist* was provided by the transitory overexpression of *Eif1* in *Eif1*-silenced clones (Figure 2A). Overexpression of *Eif1* induced high levels of *Xist* RNA in transfected cells (Figure 2B). Expression of other members of the Eif gene family was unaffected by the overexpression of *Eif1* (unpublished data), in agreement with these genes being unaffected in the *Eif1* knockdown clones (unpublished data). We conclude that altering *Eif1* expression levels leads to specific effects on *Xist* RNA levels.

### Clones Silenced for *Eif1* and *Rent1* Fail to Undergo XCI

**Figure 3:**
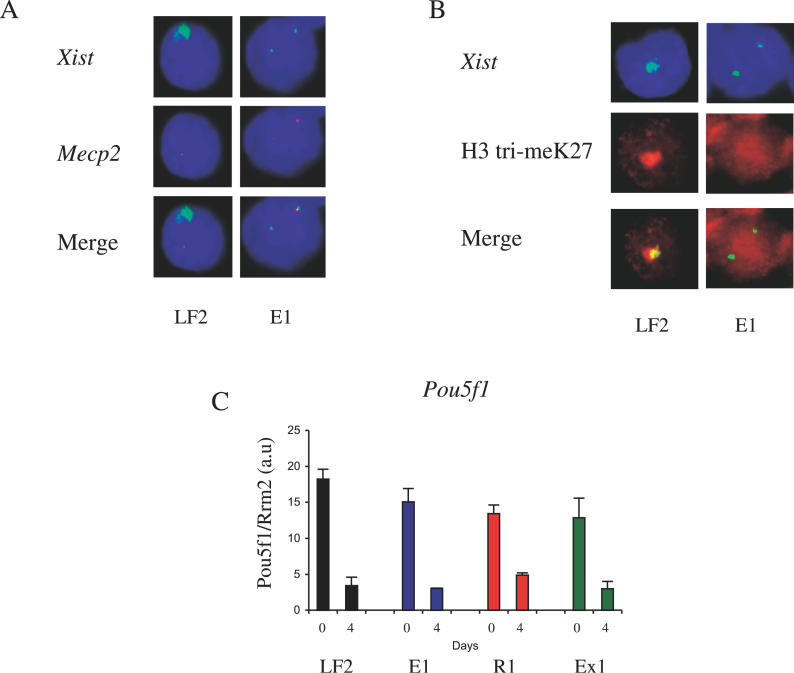
Analysis by RNA-FISH and RNA-Immunofluorescence of H3K27 Methylation Enrichment on the X Chromosome and *Pou5f1* mRNA Levels during Differentiation (A) RNA-FISH analysis of *Xist* and *Mecp2* expression at d 4 of differentiation in a control culture (LF2) and *Eif1* knockdown clone (E1), using the lambda 510 (*Xist*/*Tsix,* in green) and *Mecp2* probes (in red). The presence of a single *Xist* RNA domain corresponding to the inactive X and a pinpoint *Mecp2* signal corresponding to the active X chromosome are observed in 50% or more of cells of the control cell line (LF2). In contrast, *Xist* domains are not observed in the silenced cell line (E1), in which two *Xist/Tsix* pinpoint signals and two *Mecp2* pinpoint signals can be observed, indicating that both Xs are active. (B) Representative nuclei of differentiating ES cells hybridized with *Xist* RNA (green), and stained with antibodies to H3di/tri-meK27 [20], detected using a goat anti-mouse Alexa 680 secondary antibody (Molecular Probes, Eugene, Oregon, United States; red). In the control uninterfered cell line (LF2) the accumulation of *Xist* RNA was accompanied by enrichment of H3 di/tri-meK27 by d 4 of differentiation [20]. No enrichment of H3 tri-meK27 was observed in the interfered cell line (E1), in which 2 pinpoints of *XistTsix* are observed. (C) Quantification, using Pou5f1Up/Lo primers, of spliced *Pou5f1* RNAs at d 0 and d 4 of differentiation, in the parental female ES cell line (LF2), the clones interfered for *Eif1* (E1), *Rent1* (R1), and *Exosc10* (Ex1). Columns and bars are constructed as in Figure 1.

To investigate whether *Xist* RNA domains could be established in differentiated ES clones silenced for *Eif1* and *Rent1* despite the low level of *Xist* expressed as measured by Q-RT-PCR (Figure 1B), we performed RNA-FISH on cells differentiated for 4 d. At this stage, 50% of WT LF2 cells displayed a *Xist* domain that allowed the unequivocal identification of the inactive X chromosome. In striking contrast, no *Xist* domains could be detected in the *Eif1* and *Rent1* knockdown clones at either d 4 or even d 14 of differentiation (Figure 3A and unpublished data). The *Eif1* and *Rent1* knockdown clones maintain an X chromosome profile identical to that of undifferentiated cells with 100% of d-4 differentiated *Eif1-* and *Rent1-*silenced cells displaying two pinpoints for *Xist/Tsix* (probes lambda 510 and *Pas34* specific for *Tsix;* Figure 3A and unpublished data). Phase-contrast microscopy of differentiated *Eif1* and *Rent1* silenced ES cell cultures (data not shown) and Q-RT-PCR analysis of the differentiation marker *Pou5f1* (other name, *Oct4;* Figure 3C), a gene expressed in undifferentiated ES cell lines which is turned off during differentiation, confirmed that the *Eif1* and *Rent1* knockdown clones had been able to undergo normal differentiation.

In order to probe further the X-inactivation status of the *Eif1-* and *Rent1*-interfered clones, we explored the expression of two X-linked genes, *Mecp2* and *Chic1,* by RNA-FISH (Figure 3A and unpublished data). The presence of two pinpoint signals for *Mecp2* and *Chic1* in all knockdown clones at d 4 of differentiation indicated that both X chromosomes had remained active. Analysis of H3K27 trimethylation, a histone modification acquired by the inactive X early in the inactivation process [20], confirmed the absence of an inactive X (Figure 3B). Our results clearly implicate the *Eif1* and *Rent1* gene products in the establishment of XCI.

### *Eif1* and *Rent1* Are Involved in the Post-Transcriptional Regulation of *Xist* RNA

**Figure 4:**
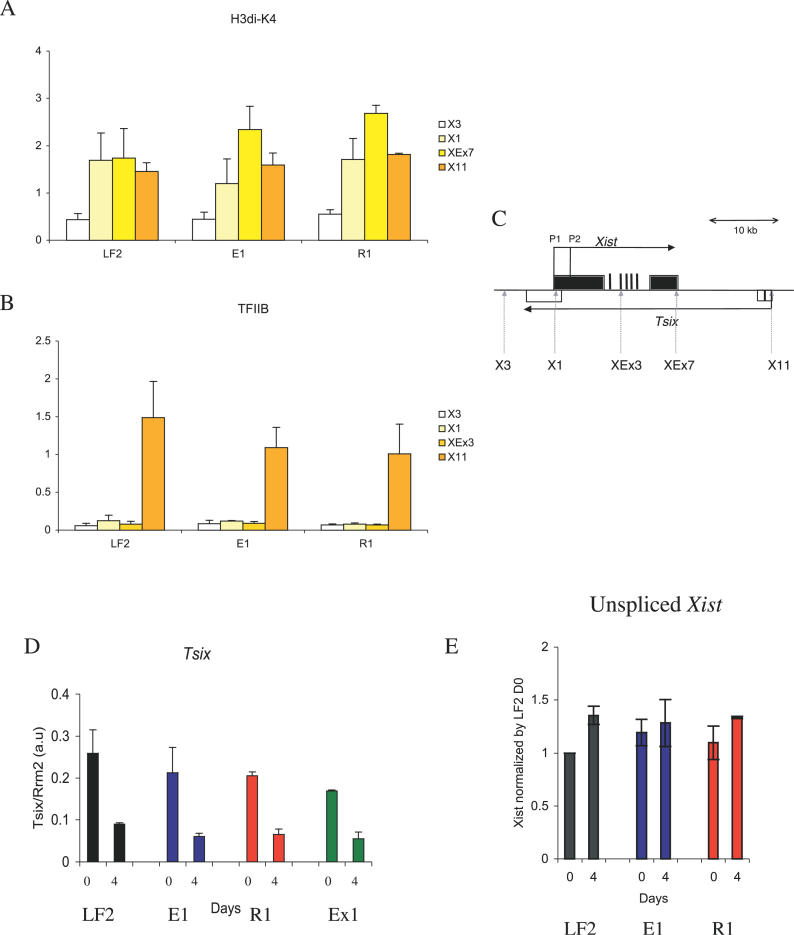
ChIP Analysis of the *Xist*/*Tsix* Region in *Eif1-* and *Rent1*-Interfered Clones and *Tsix* mRNA Levels during Differentiation (A) Analysis of H3K4 dimethylation in the *Xist*/*Tsix* region by ChIP. The graph shows the percentage of immunoprecipitation obtained after normalisation using a control position corresponding to the promoter of the X-linked gene *G6pd.* (B) ChIP analysis of TFIIB distribution in the *Xist*/*Tsix* region. The graph represents the percentage of immunoprecipitation obtained after normalisation using a control position located to the promoter of the *Arpo* gene. (C) A schematic representation of the *Xist*/*Tsix* region. Positions of the primers used in the ChIP studies are indicated (see [4]). (D) Quantification of spliced *Tsix* RNAs using exon2–exon3 primers at d 0 and d 4 of differentiation, in the parental female ES cell line (LF2) and clones knocked down for *Eif1* (E1 and E2), *Rent1* (R1 and R2), and *Exosc10* (Ex1 and Ex2). Columns and bars are constructed as in Figure 1. (E) Quantification of unspliced *Xist* transcripts by strand-specific RT-PCR using the XistIN1DLo primer for priming the RT reaction and primers XistIN1CUp/Lo for amplification. All the primers are located in intron 1 of *Xist.* Control reactions without RT, or carried out in the presence of the enzyme but without the RT primer, were also performed and shown to be negative (unpublished data).

In order to gain mechanistic insights into *Eif1* and *Rent1* function in XCI and in particular into the regulation of *Xist,* we first addressed the chromatin structure of the *Xist*/*Tsix* locus in relation to preinitiation complex recruitment at the *Xist* and *Tsix* promoters. We recently demonstrated that the *Xist*/*Tsix* locus is enriched for dimethylated H3K4 in undifferentiated ES cells, and that the deposition of this modification is dependent on *Tsix* [4]. As shown in Figure 4A, the H3K4 dimethylation profile of the locus (Figure 4C) was similar in *Eif1-* and *Rent1*-silenced cell lines and in control cells. Moreover, no significant differences in the recruitment of TFIIB, a factor that is part of the RNA polymerase II preinitiation complex, to the *Tsix* promoter were observed (Figure 4B). Taken together with the normal expression levels of *Tsix* detected by Q-RT-PCR (Figure 4D), these results indicate that *Tsix* transcription is unaffected by the reduction in *Eif1* and *Rent1* transcript levels in undifferentiated ES cells. The very low levels of TFIIB binding detected at the *Xist* promoter even in normal undifferentiated ES cells [4] (Figure 4B)—reflecting the transcriptional downregulation of *Xist* in these cells—precludes the drawing of definitive conclusions by chromatin immunoprecipitation (ChIP) analysis as to whether *Eif1* and/or *Rent1* are acting on *Xist* at the transcriptional or post-transcriptional level. To clarify this point, we have measured the levels of unspliced *Xist* RNA in undifferentiated and differentiated ES clones by strand-specific Q-RT-PCR using primers located in intron 1 of *Xist.* Strikingly, and despite the massive drop in the levels of mature *Xist* RNA transcripts in differentiated ES cells (Figure 1B), unspliced *Xist* RNA could be identified in both undifferentiated and differentiated E1 and R1 clones at levels very similar to that found in WT LF2 cells (Figure 4E). Our results strongly suggest that both *Eif1* and *Rent1* are affecting *Xist* mRNA at the post-transcriptional level.

### The NMD Pathway Is Implicated in *Xist* RNA Regulation

**Figure 5:**
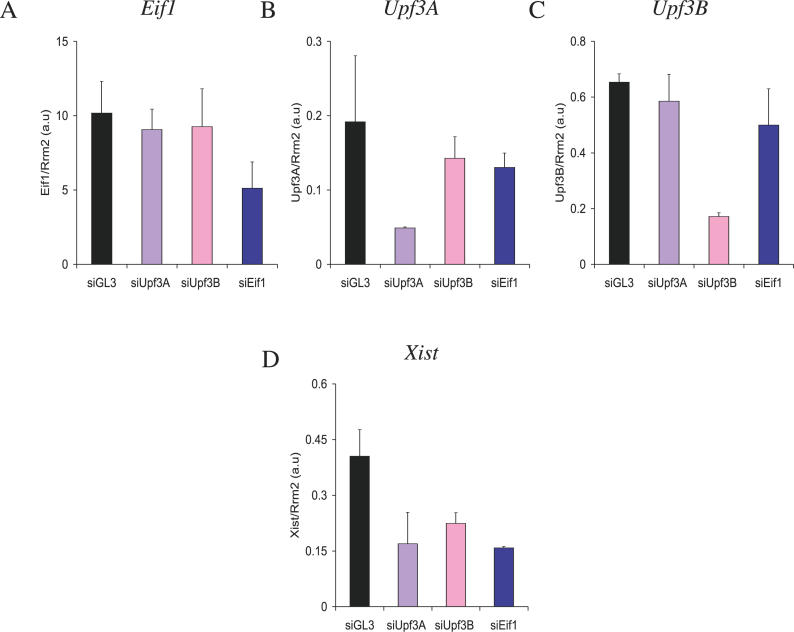
Transient Interference of *Eif1, Upf3A,* and *Upf3B* Quantification of *Eif1* (A), *Upf3A* (B), *Upf3B* (C), and *Xist* (D) RNAs using Eif1Up/Lo, Upf3AUp/Lo, Upf3BUp/Lo, and Xist exon1–exon3 primers, respectively, after 48 h of interference using siRNA siGL3, siEif1, siUpf3A, and siUpf3B. Columns and bars are constructed as in Figure 1.

The NMD pathway, which acts post-transcriptionally, is involved in mRNA degradation (for review see [21]). To further investigate a putative role of NMD in *Xist* RNA regulation, we have transiently silenced two other putative members of the pathway, *Upf3A* and *Upf3B,* using short interfering RNA (siRNA) [22]. *Upf3A* and *Upf3B* are thought to be specifically involved in the NMD pathway, unlike the *Eif1* and the *Rent1* genes, which are known to participate in processes other than NMD. After 48 h of interference, *Upf3A* and *Upf3B* were specifically downregulated in LF2 cells, compared to control cells transfected with a siRNA directed against the exogenous luciferase gene *GL3* [23] (Figure 5B and 5C). As shown in Figure 5D, undifferentiated ES cells silenced for the *Upf3A* and *Upf3B* genes (siUpf3A and siUpf3B, respectively) displayed reduced levels of *Xist* RNA compared to control ES cells (siGL3). Similar results were observed with an siRNA against the *Eif1* gene (Figure 5A and 5D). These results confirm a role for the NMD pathway in the metabolism of *Xist* RNA.

### Nuclear Exosome Pathway Is Implicated in *Xist* RNA Regulation

Recent studies have shown that the NMD pathway interacts with the exosome, which has been implicated in RNA degradation in both the nucleus and the cytoplasm [24,25]. The *Exosc10* gene, the mouse orthologue of the yeast *Rrp6* gene, has been described to be associated exclusively with nuclear exosome activity [26,27]. Based on this, we generated independent stably interfered clones for *Exosc10* (Ex1, Ex2; Figure 1A). *Xist* RNA levels are downregulated during differentiation of these clones and X-inactivation is inhibited as observed for *Eif1* and *Rent1* (Figure 1B). Based on these results, it appears likely that a nuclear-specific mRNA degradation pathway is an essential player in the X-inactivation process.

### Upregulation of *Xist* cDNA Transgene in Silenced Stable Clones

**Figure 6:**
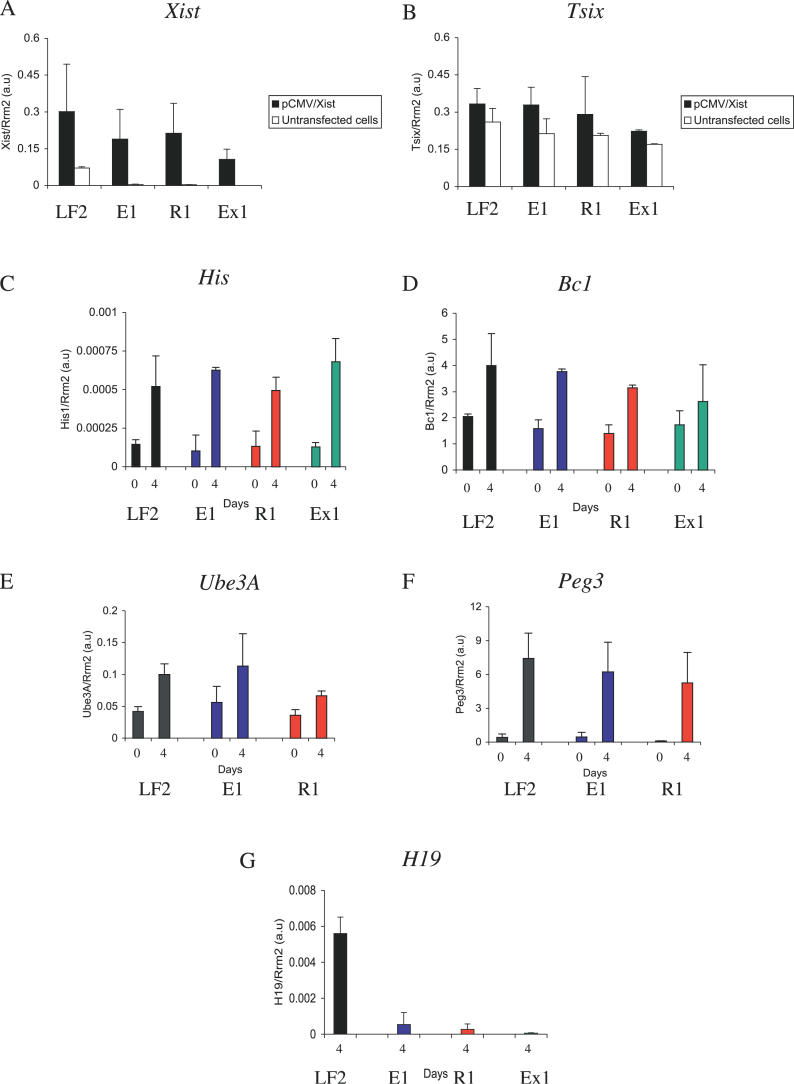
Overexpression of *Xist* cDNA Transgene and Impact of Interference on ncRNAs and Imprinted RNAs (A–B) Quantification of *Xist* and *Tsix* RNAs using Xist exon1–exon3 and Tsix using exon2–exon3 primers in the parental female ES cell line (LF2) and knocked down clones for *Eif1* (E1), *Rent1* (R1), and *Exosc10* (Ex1), and in the same cells 48 h after transfection with *Xist* cDNA transgene. Quantification of *His1* (C), *Bc1* (D), *Ube3A* (E), *Peg3* (F), and *H19* (G) RNAs using His1Up/Lo, Bc11Up/Lo, Ube3AUp/Lo, Peg3Up/Lo, and H19Up/Lo primers respectively, at d 0 and d 4 of retinoic acid–induced ES cell differentiation. Columns and bars are constructed as in Figure 1.

Our finding that unspliced *Xist* is not affected by the downregulation of *Eif1* and *Rent1* strongly suggested that only spliced forms of *Xist* are affected. To investigate whether this reflects a change in the stability of spliced *Xist* transcripts, we have transiently transfected *Xist* cDNA constructs [28] into WT ES cells and into ES cells silenced for *Eif1, Rent1,* and *Exosc10.* As shown in Figure 6A and 6B, we observed a strong upregulation of *Xist* RNA levels in all cells, in the absence of marked variation for *Tsix* RNA levels. Our results suggest that the *Xist* exogenous cDNA is unaffected in the interfered clones, implying rather that it is at the splicing step that *Xist* metabolism is perturbed in the interfered clones. To explore further a link between the transcription, splicing step, and the effects we had seen in *Xist* metabolism, we first treated cells (T1 and E1 clones) differentiated for 4 d with actinomycin D for 0–8 h to inhibit transcription, then isolated RNA at 2-h timepoints, and quantified by real-time RT-PCR the *Xist* RNA using both strand-specific and exon–exon primers. Half-lives in the control and interfered clone were similar, suggesting that the degradation pathway does not affect *Xist* levels in the absence of active transcription. Levels of *Xist* pre-mRNA also had similar half-lives in both the T1 and E1 clones (Figure S1). Taken altogether, our results suggest that the *Xist* processing interfered with in the *Eif1* knockdown clones is closely associated with the splicing process occurring during active transcription.

### Analysis of Other Noncoding and Imprinted RNAs

Our results pose the question as to the eventual involvement of a nuclear mRNA degradation pathway in the regulation of other noncoding RNAs (ncRNAs) or imprinted genes. To probe this, we have analysed the expression of several other ncRNAs and/or imprinted genes (Figure 6C–6G). Neither *His1* and *Bc1* ncRNAs nor the *Ube3A* and *Peg3* imprinted genes were affected by the downregulation of *Eif1, Rent1,* and *Exosc10* (Figure 6C–6F). In contrast, expression of the *H19* gene, which occurs only in differentiated ES cells, was highly inhibited (Figure 6G). We conclude that *Eif1, Rent1,* and *Exosc10* are not part of a general mechanism for regulating ncRNAs and imprinted genes, but rather affect a highly restricted subset of such genes.

## Discussion

In this manuscript, we have demonstrated that mRNA degradation pathways are involved in the regulation of *Xist* metabolism. Using RNA interference, we generated stable female ES cell lines for the *Eif1, Rent1,* and *Exosc10* genes. Clones silenced for each of these genes fail to upregulate *Xist* during cellular differentiation, and X-inactivation establishment is aborted.

Measurement of the levels of spliced and unspliced *Xist* in *Eif1-* and *Rent1*-inhibited clones showed that unlike spliced *Xist* transcripts, unspliced *Xist* transcripts were maintained at similar levels in the silenced clones to those of the control WT female ES cell lines both before and after differentiation (Figure 4E). Since there is no evidence for alternative promoter use in *Xist* transcription in ES cells, we conclude that *Eif1* and *Rent1* cannot be affecting *Xist* metabolism via transcription initiation itself but are acting at the post-transcriptional level. Moreover, the dramatic decrease in spliced *Xist* RNA transcript levels occurs in the absence of any detectable effect on *Tsix* levels as analysed by Q-RT-PCR. Our results underline the specificity of this RNA degradation control for *Xist*, since many other ncRNAs are unaffected.

The *Rent1* gene, unlike the *Upf3A* and *Upf3B* genes, participates not only in the NMD but also in nonsense-mediated altered splicing [29], Staufen1-mediated decay [30], and the efficiency of alternative splicing [31]. To provide further support for the notion that the NMD pathway is involved in the post-transcriptional control of *Xist* transcript levels, we therefore transiently interfered two additional genes, *Upf3A* and *Upf3B*. The downregulation of *Xist* RNA transcript levels in cells silenced for the *Rent1, Upf3A,* and *Upf3B* genes suggests that the effects on *Xist* RNA metabolism are being mediated via the NMD pathway.

To clarify whether the degradation pathway(s) is/are acting into the nucleus, we generated clones silenced for the *Exosc10* gene, which is specific to the nuclear exosome pathway [26,27]. Silenced clones for the *Exosc10* gene showed dramatically reduced levels of *Xist* RNA. Based on a parsimonious interpretation of our results, we therefore propose that a nuclear pathway, involving either all or many components of the NMD pathway, participates either directly or indirectly in the regulation of *Xist* RNA and XCI. We hypothesise that these effects are based around interactions occurring between the NMD machinery and elements of the splicing machinery during transcription/elongation-linked activities. The NMD pathway is known to interact directly with elements of the splicing machinery and the process of splicing itself [21]. Similarly, there is increasingly well-supported experimental evidence for the functional coupling of the transcription and splicing processes, with many active RNA polymerase II elongation complexes being found associated with splice complex and exosome components (for review see [32]). Recent experiments have shown that upregulation of *Xist* RNA is a critical step in the establishment of the X inactivation process, and that this is regulated transcriptionally [4,5] rather than via stabilization of the *Xist*-spliced form(s) as previously hypothesised [1,2]. Since in the interfered clone only spliced forms of *Xist* are downregulated and the stability of unspliced and spliced forms of *Xist* RNA and the level of unspliced *Xist* are comparable in control and interfered clones (T1 and E1), it is likely that it is a transcription-coupled splicing step itself that is being affected in our clones.

While further work will be necessary to consolidate our understanding of the precise mechanisms involved, and to elucidate whether the NMD components are acting directly or indirectly, our results open up another dimension to our knowledge of the pathways, that concur to ensure that levels of *Xist* are stringently controlled both prior to and at the onset of the X-inactivation process [3–5] (Navarro et al., unpublished data). Both the levels of *Xist* transcript and the spatial distribution of these transcripts are likely to be critical to this process.

## Materials and Methods

### Culture and in vitro differentiation of ES cells.

Female LF2 ES cell lines were cultured in Dulbecco's Modified Eagle Media (DMEM; Invitrogen, Carlsbad, California, United States), containing 15% fetal calf serum (FCS; Bio West), 1,000 U/ml LIF (Chemicon, Temecula, California, United States), 0.1 mM 2-mercaptoethanol (Invitrogen), 0.05 mg/ml streptomycin (Invitrogen), and 50 U/ml penicillin (Invitrogen) on a gelatin-coated support in the absence of feeder cells. Differentiation was induced by adding 100 nM all *trans* retinoic acid (Sigma, St. Louis, Missouri, United States) to LIF-free DMEM and 10% FCS medium for the first 3 d of differentiation. The culture medium was changed daily. All cells were grown at 37 °C in 8% CO_2_.

### Construction of RNA interference vectors.

The pSUPER-puro vector (OligoEngine, http://www.oligoengine.com) was used according to the manufacturer's instrutions. Sequences coding for short hairpin RNAs were inserted as double-stranded oligos into pSUPER-puro using the BglII and HindIII sites as described previously [19]. The target sequence for the *Eif1* gene was 5′-GGACGATCAGCTGAAGGTT-3′, the two target sequences for the *Rent1* gene were 5′-GCGCACCGCTGAGAGAGAA-3′ (hairpin 1) and 5′-GCAGCCAATGTGGAGAAGA-3′ (hairpin 2), and the two target sequences for the *Exosc10* gene were 5′-GGAGCCTCAAGGCATCATA-3′ (hairpin 1) and 5′-GCCCAGAACATAATGCAGT-3′ (hairpin 2). All target sequences have been blasted using National Center for Biotechnology Information software and shown to be specific for the gene of interest (http://www.ncbi.nlm.nih.gov/BLAST). The T1 clone is a control generated by integration of an empty pSuper-puro vector into the LF2 cell line. To test for an adverse nonspecific effect of RNAi mechanisms on *Eif1, Rent1,* and *Exosc10* expressions, we generated stable LF2 cells interfered for the *Rasa1* gene (Ra1 and Ra2) [33]. Transfection of different plasmids was carried out using Lipofectamine 2000 (Invitrogen). Stable integrants of pSuper-puro plasmids were selected on puromycin containing medium while stable integrants of the *Rasa1* plasmid were selected on G418-containing medium.

### Overexpression of *Xist* and *Eif1.*

The pTriEx-1.1 Neo vector (Novagen, Madison, Wisconsin, United States) was used according to manufacturer's instructions. The open reading frame (ORF) of the *Eif1* gene was amplified by PCR using the following primers:

5′-AATGGTCTCGCATGTCCGCTATCCAGAACCTCCAGAACCTCCACT-3′ and

5′-CACCTGAGGTTAAAACCCATGAACCTTCAGCTGATCGTCCTTAG-3′, which contain BsaI and Bsu36I enzyme restriction sites respectively used for cloning. Plasmids pCMV-Xist [28] and pTriEx-Eif1 were transfected using Lipofectamine 2000 (Invitrogen). *Eif1* and *Xist* overexpressing cells were selected for on G418-containing medium for 4 d and 48 h, respectively.

### Real-time quantitative RT–PCR.

Total RNA was prepared using RNable (Eurobio, Courtaboeuf, France) and verified by electrophoresis. random-primed reverse transcription (RT) was carried out using the Superscript II reverse transcriptase (Invitrogen). Control reactions without enzyme were systematically performed. cDNAs were analyzed by real-time PCR using the SYBR Green Universal Mix and an ABI Prism 7700 (Perkin-Elmer Applied Biosystems, Wellesley, California, United States). Each PCR was run in triplicate to control for PCR variation. *Rrm2* transcript levels were used for normalization [9]. All primer sequences are available on request. For strand-specific RT, we used the ThermoScript reverse transcriptase (Invitrogen).

### Immunofluorescence and RNA-FISH analysis.

The preparation of nuclei for RNA-FISH, hybridization, and washes were performed as described [34] using DNA probes labelled by nick translation with Spectrum Red or Green dUTP (Vysis, Downers Grove, Illinois, United States). For immunofluorescence experiments performed after RNA-FISH, preparations were rinsed in phosphate-buffered saline (PBS), blocked in 0.5% bovine serum albumin (BSA) (NEB) in PBS for 15 min at room temperature, incubated with mouse monoclonal H3 di/tri-meK27 antibody at 1/100 (7B11) [20] for 45 min at room temperature, then rinsed in PBS and incubated with Texas red–conjugated goat anti-mouse secondary antibody for 45 min at room temperature. All antibodies were diluted in PBS/0.5% BSA. DNA was counterstained for 2 min with DAPI (0.2 mg/ml). Samples were mounted in 90% glycerol, 10% 1× PBS, 0.1% p-phenylenediamine (pH 9) (Sigma). A Zeiss Axioplan fluorescence microscope equiped with a Quantix CCD camera (Photometrix, Melbourne, Australia) and SmartCapture 2 software (Digital Scientific, Cambridge, United Kingdom) were used for image acquisition. All the probes used in this study have been described previously [35,36]. The results were obtained when cultures were examined after 4 and 14 d of differentiation (data not shown).

### ChIP.

Immunoprecipitations were performed using a dimethylated H3 Lys-4 antibody (Upstate Biotechnology, Lake Placid, New York, United States) and TFIIB antibody (C-18; Santa-Cruz Biotechnology, Santa Cruz, California, United States), as described previously [4].

### Transitory tranfection of siRNA.

siRNAs *GL3* [23], *Upf3A, Upf3B* (Qiagen, Valencia, California, United States), and *Eif1* (5′-GGTTCATGGGTTTTAAGTGdTdT-3′) were transfected using Lipofectamine 2000 (Invitrogen). Levels of interference were monitored by Q-RT-PCR as described previously.

### *Xist* and *Tsix* half-life assay.

ES cells (T1, E1 clones; 10^5^) were dispensed into each well of a six-well plate and differentiated, using retinoic acid, during 4 d. Fresh media containing 5 μg/ml actinomycin D was then added. At each time point, media was aspirated and total RNA was prepared using RNable (Eurobio). RNA levels were quantified as described in Materials and Methods by Q-RT-PCR.

## Supporting Information

Figure S1 Low Levels of *Xist*-Spliced Forms Are Not Mediated by a Transcription-Independent RNA Degradation Mechanism *Xist* pre-mRNA half-life(s) were measured by strand-specific, real-time RT-PCR after actinomycin D treatment. Cell culture experiments were performed in replicate, and PCR cycling reactions in triplicate. Points represent averages from duplicate experiments ± standard error of the mean.
(24 KB PDF)

### Accession Numbers

The GenBank (http://www.ncbi.nlm.nih.gov/Genbank) accession numbers for the genes discussed in this paper are *Eif1* (20918), *Rent1* (19704), *Exosc10* (50912), *Xist* (213742), *Tsix* (22097), *Pou5f1* (18999), *Upf3A* (67031), *Upf3B* (68134), *His1* (110153), *Bc1* (12031), *Ube3a* (22215), *Peg3* (18616), *H19* (14955), *Rasa1* (218397), and *Rrm2* (20135).

## Abbreviations

ChIP: chromatin immunoprecipitation
ES: embryonic stem
ncRNA: noncoding RNA
NMD: nonsense-mediated decay
ORF: open reading frame
RNA-FISH: RNA–fluorescence in situ hybridization
siRNA: short interfering RNA
XCI: X-chromosome inactivation
WT: wild-type
