# Supplementary material for: Nuclear mRNA Degradation Pathway(s) Are Implicated in Xist Regulation and X Chromosome Inactivation
Source: PLoS Genet. 2006 Jun 16;2(6):e94. doi: 10.1371/journal.pgen.0020094 (PMC1479048; doi:10.1371/journal.pgen.0020094)
Supplement: Supplementary file 1 [file pgen.0020094.sg001.pdf]

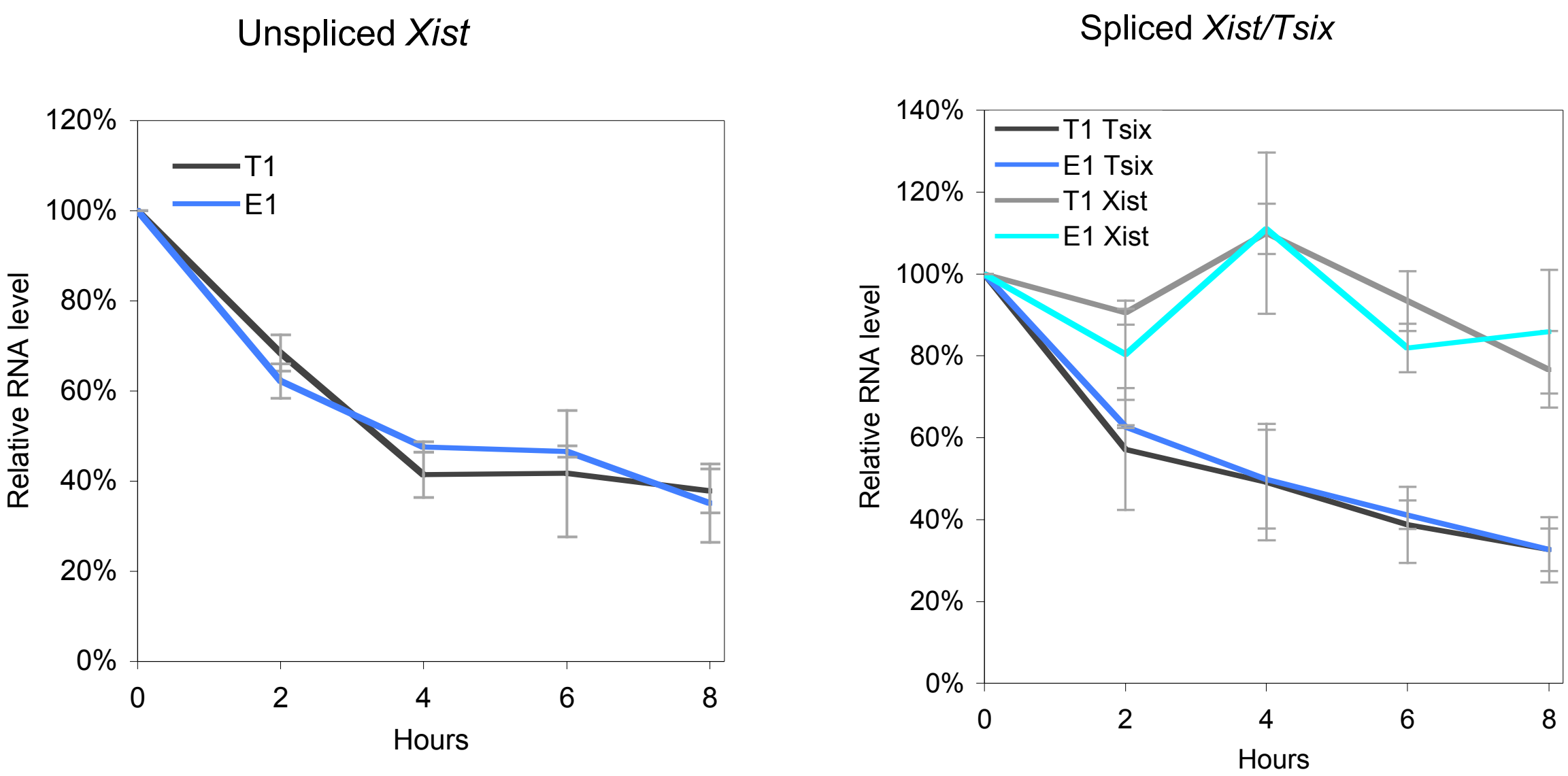

Unspliced *Xist*
120%
100%
80%
60%
40%
20%
0%
Relative RNA level
T1
E1
0
2
4
6
8
Hours
Spliced *Xist/Tsix*
140%
120%
100%
80%
60%
40%
20%
0%
Relative RNA level
T1 Tsix
E1 Tsix
T1 Xist
E1 Xist
0
2
4
6
8
Hours
